# Offspring sex impacts DNA methylation and gene expression in placentae from women with diabetes during pregnancy

**DOI:** 10.1371/journal.pone.0190698

**Published:** 2018-02-22

**Authors:** Jacqueline Alexander, April M. Teague, Jing Chen, Christopher E. Aston, Yuet-Kin Leung, Steven Chernausek, Rebecca A. Simmons, Sara E. Pinney

**Affiliations:** 1 Division of Endocrinology and Diabetes, The Children’s Hospital of Philadelphia, Philadelphia, Pennsylvania, United States of America; 2 Department of Pediatrics, CMRI Metabolic Research Program, University of Oklahoma Health Sciences Center, Oklahoma City, Oklahoma, United States of America; 3 Harold Hamm Diabetes Center, University of Oklahoma Health Sciences Center, Oklahoma City, Oklahoma, United States of America; 4 Department of Environmental Health, University of Cincinnati College of Medicine, Cincinnati, Ohio, United States of America; 5 Division of Neonatology, The Children’s Hospital of Philadelphia, Philadelphia, Pennsylvania, United States of America; 6 Department of Pediatrics, Perelman School of Medicine at the University of Pennsylvania, Philadelphia, Pennsylvania, United States of America; University of Southampton, UNITED KINGDOM

## Abstract

**Aims/Hypothesis:**

We hypothesized that diabetes during pregnancy (DDP) alters genome-wide DNA methylation in placenta resulting in differentially methylated loci of metabolically relevant genes and downstream changes in RNA and protein expression.

**Methods:**

We mapped genome-wide DNA methylation with the Infinium 450K Human Methylation Bead Chip in term fetal placentae from Native American and Hispanic women with DDP using a nested case-control design (n = 17 pairs). RNA expression and protein levels were assayed via RNA-Seq and Western Blot.

**Results:**

Genome-wide DNA methylation analysis revealed 465 CpG sites with significant changes for male offspring, 247 for female offspring, and 277 for offspring of both sexes (p<0.001). Placentae from female offspring were 40% more likely to have significant gains in DNA methylation compared with placentae from male offspring exposed to DDP (p<0.001). Changes in DNA methylation corresponded to changes in RNA and protein levels for 6 genes: PIWIL3, CYBA, GSTM1, GSTM5, KCNE1 and NXN. Differential DNA methylation was detected at loci related to mitochondrial function, DNA repair, inflammation, oxidative stress.

**Conclusions/Interpretation:**

These findings begin to explain mechanisms responsible for the increased risk for obesity and type 2 diabetes in offspring of mothers with DDP.

## Introduction

Diabetic pregnancy induces marked abnormalities in glucose homeostasis and insulin secretion in the fetus resulting in abnormal fetal growth [1]. Population-based studies have demonstrated that offspring of diabetic mothers have an increased risk for obesity, glucose intolerance, and type 2 diabetes especially in Native American populations [2, 3]. It has been proposed that early exposure to hyperglycemia and elevated insulin levels may lead to malprogramming of the fetus leading to the subsequent development of diabetes and obesity. The link between an adverse intrauterine environment and the later development of diabetes and obesity has been observed in offspring of diabetic pregnancies, but the molecular mechanisms are unknown [3, 4]. Epigenetic modifications of the genome including DNA methylation, provide a plausible mechanism that allows for permanent propagation of gene activity states from one generation of cells to the next [5]. Epigenetics refers to non-sequence based structural changes that can alter gene expression and can be modified by environmental factors and may serve as biological memory of an aberrant intrauterine environment [6]. With the increased prevalence of obesity and diabetes, it is critical to examine the potential role of epigenetic programming in the development of these diseases.

The placenta is a complex organ and is essential in regulating fetal growth. The altered metabolic milieu of maternal diabetes is associated with changes in placental glucose transport, increased amino acid transport, and changes in fatty acid uptake and metabolism [7–10]. The changes in placental nutrient transport associated with diabetes during pregnancy (DDP) have significant effects on the developing fetus, indicating that the placenta plays a critical role in fetal programming.

The aim of our study was to investigate whether exposure to DDP alters genome-wide DNA methylation in the placenta resulting in differentially methylated loci of metabolically relevant genes and downstream changes in RNA and protein expression. To test this hypothesis we mapped genome-wide DNA methylation with the Infinium 450K Human Methylation Bead Chip using a nested case-control design from a cohort of Native American and Hispanic women with DDP, followed by RNA-Seq to quantify changes in RNA expression and Western Blot to quantify corresponding changes in protein levels.

## Materials and methods

### Patient characteristics

Native American and Hispanic women experiencing a pregnancy complicated by diabetes (DDP, n = 17) including patients with both gestational (n = 14) or pre-existing Type 2 diabetes (n = 3) diagnosed by American Diabetes Association criteria [11], were selected from a larger cohort of maternal/offspring dyads with exposure to DDP [12, 13]. The participants with DDP in this study were matched 1:1 with women experiencing a normal pregnancy (control; n = 17). A nested case-control design was used to decrease variance between groups. Matching criteria included offspring sex, maternal race/ethnicity, maternal age and gestational age at birth. Exclusion criteria included preterm delivery, congenital birth defects, birth asphyxia, congenital infection, offspring metabolic disease, or maternal complications (i.e. preeclampsia). All mothers provided written informed consent in accordance with the Institutional Review Boards of the Chickasaw Nation (Ada, Oklahoma), Choctaw Nation of Oklahoma (Talihina, OK) and the University of Oklahoma Health Sciences Center (Oklahoma City, OK), which approved the study.

### Genome wide DNA methylation analysis

Placentae were obtained and dissected within 1 hour of delivery. A 3 cm diameter core was taken from the fetal surface through to the maternal surface halfway between the umbilical cord and the placental margin. Only the fetal placental portion was studied after it was washed in saline and stored at -80C. Please see S1 Text for additional details.

Genomic DNA was isolated (DNeasy, Qiagen) and processed by the University of Cincinnati Genomics, Epigenomics and Sequencing Core including bisulfite conversion for Illumina’s Infinium 450K Human Methylation Bead Chip. The methylation status, *β*, was calculated at each probe site as *β* = M/(M+U), where M = methylated and U = unmethylated. Differential methylation was calculated for each sample pair and averaged across all pairs for each probe site to determine the absolute value of the difference in methylation between DDP and control (AVDM). CpGs on the X chromosome were excluded based on complications from X inactivation and the high proportion of tandem repeat DNA [14]. Genes with differential methylation at multiple probe sites were validated with bisulfite pyrosequencing or Mass Array Epityper. (S1 Text and S1 Table).

### RNA-Seq

RNA expression was assessed via Cofactor Genomics’ Precision-Seq RNA-Seq (St. Louis, MO) (Please see S1 Text for additional details). Six matched pairs of fetal placentae were analyzed, 3 pairs per offspring sex and 3 pairs per race/ethnicity (3 female offspring pairs (2 Hispanic and 1 Native American) and 3 male offspring pairs (1 Hispanic and 2 Native American)). An average of 38 million reads per sample were obtained. A total of 74,909 mRNA transcripts were studied, and the expression sum (a representation of read count), fold change (DDP versus control), q-value, and p-value were calculated for each transcript. Transcripts of interest were identified using criteria including expression sum > 5, fold change >1.5, and p-value < 0.05. Please see S1 Text for additional details.

### Protein levels

Confirmatory studies on protein abundance were conducted for genes in the DNA methylation and/or RNA-Seq data sets as a method to validate DNA Methylation and RNA-Seq results. Protein lysate was isolated with nuclear cell extraction buffer (Invitrogen, Carlsbad, CA) containing protease inhibitors (1mM PMSF, ThermoScientific Protease Inhibitor Cocktail) and subjected to SDS-PAGE (Life Technologies, Carlsbad, CA) and 30ug total protein were loaded per well. Membranes were blocked with 5% milk in 1X TBST, and incubated with primary antibodies (1:20,000) (PIWIL3:Thermo Scientific Pierce, PAS-21052; CYBA: Santa Cruz, sc-20781; GSTM1: Santa Cruz, sc-133641; GSTM5: Abcam, ab154018; KCNE1: Abcam, ab65795; NXN” Abcam, ab118301; ALG1: Novis Biologicals, H00056052020D01P; BCL2: Santa Cruz, sc-492; SPRY1: Santa Cruz, sc-30048; ARNT: Sigma Aldrich, SAB4501787; MTHFDL1: Santa Cruz, sc-367843). Anti-rabbit IgG, HRP-linked secondary antibody was applied (1:10,000). Membranes were developed using Amersham ECL Western Blotting Detection Kit (GE Healthcare and Life Sciences) with an Alpha Innotech FluorochemQ imager. Band density was assessed using ImageJ and normalized to β-actin (Santa Cruz, sc-10731) or cofilin (Santa Cruz, sc-33779) depending on molecular weight of the protein of interest.

### Statistical analysis

Raw methylation data were normalized using Illumina's Genome Studio and beta values were exported using R platform [15] and Bioconductor [16]. The distribution of beta-values was homogeneous and similar to the expected distribution. Correlations between all sample pairs were very high (>0.95). A paired two-sample t-test was performed at each probe site to estimate the methylation change and statistical significance. Differences in distributions between groups were analyzed using chi-squared tests or Fisher exact tests. P-values were corrected for the false discovery rate (FDR) by the method of Benjamini-Hochberg. RNA-Seq analysis included quality control, alignment, clustering, normalization, and expression comparison. P-values were calculated between the means of each pair of replicate groups using a Welch’s t-test corrected for FDR by the method of Benjamini-Hochberg. Please see S1 Text for additional details. For the studies of differences in offspring sex, comparison of proportions were performed against a 50/50 ratio, and the z-test statistic was used. Statistical calculations used SPSS (Windows, 20.0. 2011. Armonk, NY).

### Ingenuity pathway analysis

Differentially methylated and expressed genes were analyzed using Ingenuity Pathway Analysis (IPA) software (Ingenuity Systems, Redwood City, CA) to assess gene ontology enrichment and identify pathways of biological significance. Lists of differentially methylated genes were used for *all*, male, and female offspring analyses. Additional IPA analyses were performed for mRNA expression for *all*, male, and female offspring pairs. The hypergeometric test identified statistically enriched pathways and gene ontology categories.

## Results

### Clinical characteristics of study participants

**Fig 1:**
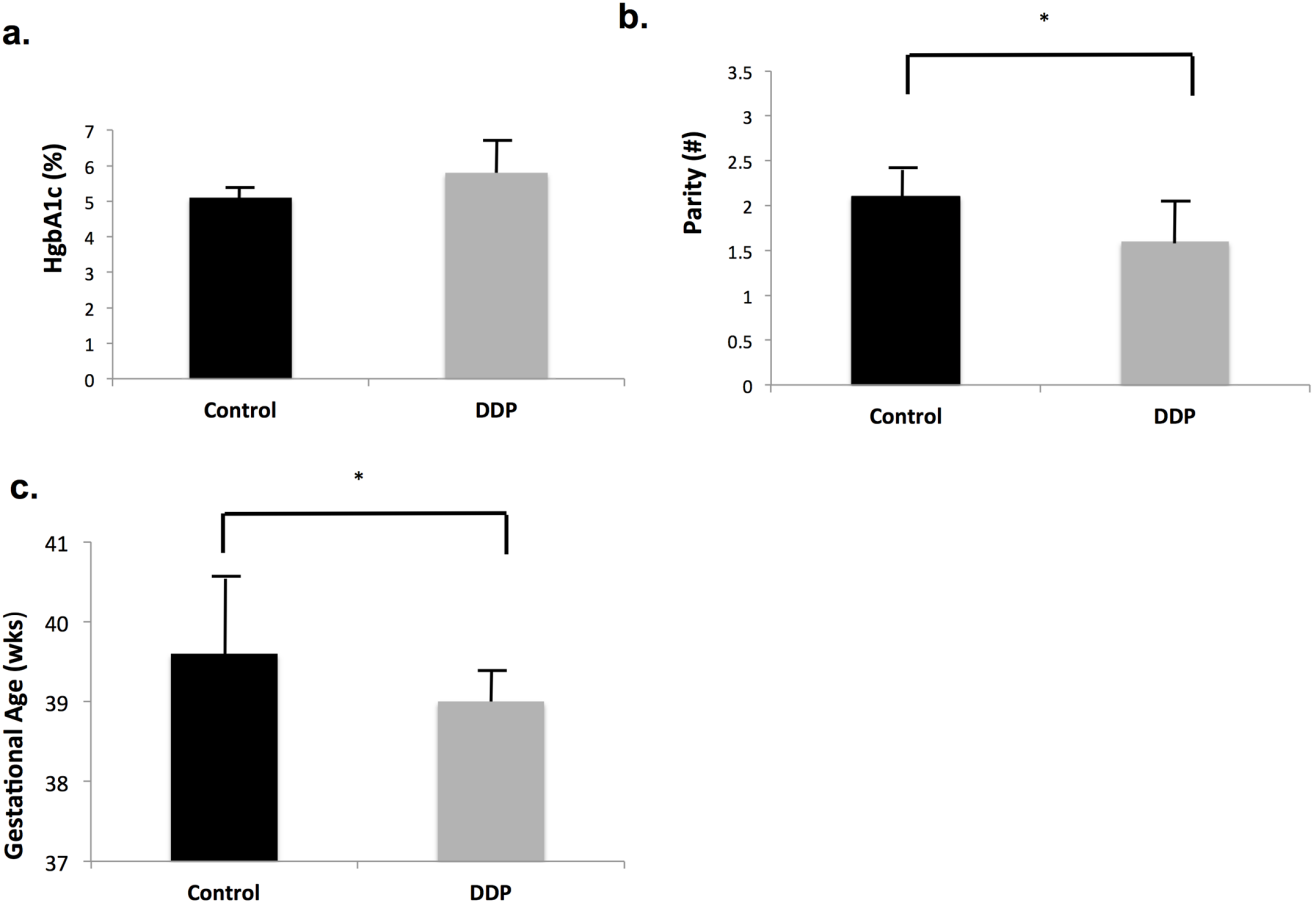
Maternal and infant characteristics. a) HgbA1C, b) Parity c) Gestational Age (weeks). *p<0.05. (n = 17 pairs).

Of the 17 matched pairs of placentae, 8 were from male offspring and 9 from female offspring. Additionally, 8 pairs of mothers were Hispanic (5 female and 3 male offspring pairs) and 9 were Native American (4 female and 5 male offspring pairs). Maternal diabetes was well controlled with diet (n = 4), insulin (n = 6) or glyburide (n = 7), as evidenced by an average HgbA_1c_ approaching that of controls. There were no significant differences in maternal age, body mass index (BMI) or pre-pregnancy weight but mothers with DDP had lower parity (*p* = 0.0053) (Table 1, Fig 1). Although all had term gestations, the gestational age at birth was 0.6 weeks (4.2 days) less for DDP infants (*p* = 0.01). No difference in birth weight was detected.

**Table 1:** Maternal and infant characteristics.

	Control (n = 17)	DDP (n = 17)	p
Maternal age	28.9 ± 4.5	28.5 ± 4.6	0.1
Parity	2.1 ± 1.1	1.6 ± 1.1	0.0053*
Pre-pregnancy weight (kg)	80.6 ± 22.1	80.1 ± 17.1	0.93
Pre-pregnancy BMI	31.8 ± 5.7	29.2± 6.9	0.27
Hgb_A1c_ (%) [mmol/mol]**	5.1 ± 0.2 [32 ± 1.3]	5.8 ± 1 [40 ± 6.9]	0.08
Gestational age at birth (wks)	39.6 ± 1	39 ± 0.4	0.010*
Birth weight (kg)	3.3 ± 0.4	3.4 ± 0.5	0.59

Data are presented as mean ± SD.

* p≤0.01,

** measured at 37 weeks gestation

### Genome wide DNA methylation

**Fig 2:**
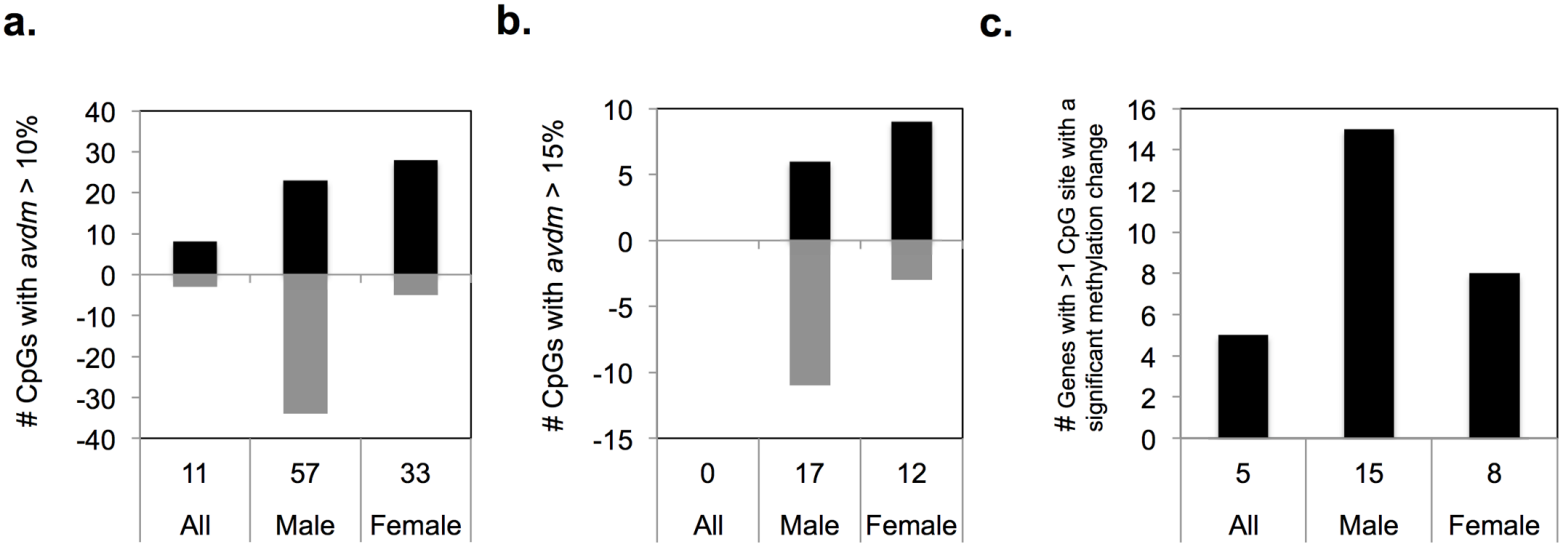
CpG sites with significant changes in DNA methylation. CpG sites with (a) *AVDM* > 10% and (b) *AVDM* > 15% and p<0.001. (a and b) Black indicates methylation increase in DDP. Gray region indicates methylation loss in DDP. (c) Genes with > 1 CpG site with significant change in DNA methylation (p<0.001).

Analysis of placental samples from male offspring revealed 57 CpG sites with AVDM ≥ 10% and 17 CpG sites with AVDM ≥ 15% and p<0.001. In female offspring pairs there were 33 CpG sites with AVDM ≥ 10% and 12 CpG sites with AVDM ≥ 15% (Fig 2). However, for the analysis of *all* pair analysis (n = 17), there were only 11 CpG sites with AVDM ≥ 10% and statistically significant changes in DNA methylation (defined as p<0.001). Thus, there was a statistically significant difference in the number of CpG sites identified with changes in DNA methylation at the ≥ 10% and ≥15% thresholds in the separate male and female analyses compared to *all* pairs (p<0.0001), but no difference in the distribution of AVDM (AVDM <10%, 10–15%, or >15%*)* in the male paired analysis versus the female paired analysis (*p* = 0.49). There were no CpG sites identified with q<0.05 after the Benjamini-Hochberg correction for multiple hypothesis testing was applied.

There were 28 genes identified with more than one differentially methylated CpG site (*p*<0.001), 5 genes in the analysis of *all* pairs, 15 in the male offspring analysis and 8 in the female offspring analysis (Fig 2c). Genes with >1 differentially methylated CpG site are of particular interest, as this could reflect an increase in the likelihood of biologically significant downstream expression changes. Finally, the total number of differentially methylated CpG sites was greater in the analysis of pairs with male offspring (n = 465) compared to those identified in the female pair offspring analysis (n = 340) (*p*<0.05).

Each analysis identified CpG sites with significant increases and decreases in DNA methylation (Fig 2). Specifically, 84% of all CpG sites with significant changes in DNA methylation (AVDM ≥ 10% and *p*<0.001) had a gain of DNA methylation in placentae from female offspring while only 40% of CpG sites with significant changes in DNA methylation (AVDM ≥ 10% and *p*<0.001) resulted in a gain of methylation in placentae from male offspring (*p*<0.0001) (Fig 2a).

**Fig 3:**
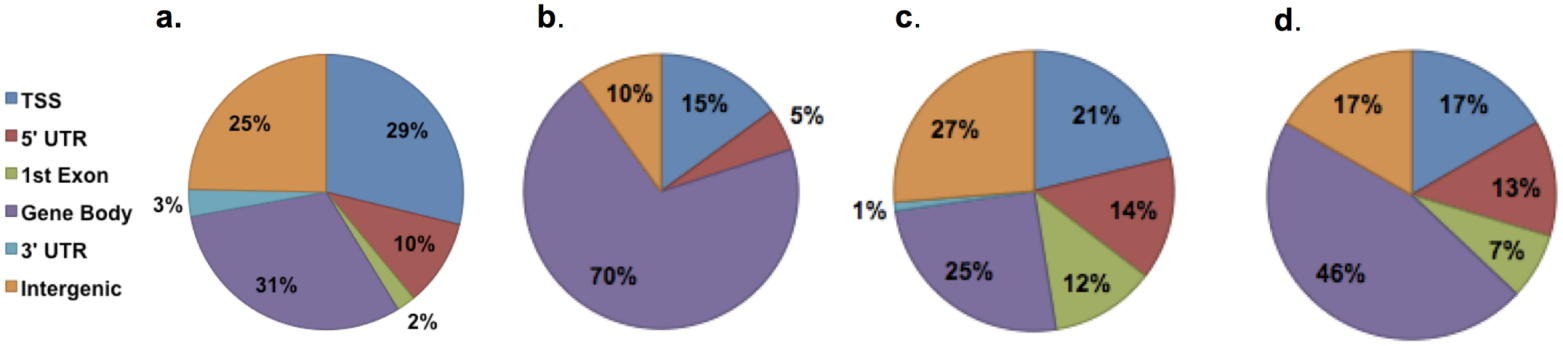
Gene regulatory regions of probe sites included on the Infinium 450K Human Methylation Bead Chip. TSS = Translational Start Site; UTR: Untranslated Region. a: Reference distribution of all probe sites included on the array. b, c and d: Distribution of probe site with *AVDM* > 10% and p< 0.001. b: *All* pairs c. Male offspring pairs. d. Female offspring pairs.

We considered CpG sites with AVDM ≥ 10% and p<0.001 to have a greater probability of eliciting significant changes in gene expression and thus focused subsequent analyses on these CpG sites. The distribution of gene regulatory regions for CpGs with significant changes in DNA methylation is presented in Fig 3. Fig 3a depicts the reference distribution of all CpG sites mapped on the Infinium 450K methylation array [17]. CpG sites are assigned to functional regions of the gene including promoter regions (transcriptional start site (TSS), 5’ UTR, and 1^st^ exon), gene body, 3’ UTR, and intergenic regions (Fig 2) [17]. Fig 3b, 3c and 3d show the distribution of the regions of the gene for CpG sites with significantly altered DNA methylation after DDP exposure. In the *all* pair analyses, CpG sites with altered DNA methylation were enriched in the gene body compared to the reference distribution (p = 0.012). In the separate analyses of male pairs and female pairs, the CpG sites with significantly altered DNA methylation were enriched for locations in the 1^st^ Exon (12% and 7% respectively) compared with the reference distribution (2%) (Male *p*<0.0001; Female *p*<0.0047). These data suggest that DDP preferentially alters DNA methylation within the first exon, and that sex of the offspring has a strong effect on which specific CpG sites are affected.

Table 2 is a summary of the CpG sites with the greatest absolute change in DNA methylation for the 3 analyses conducted. For the analysis of *all* pairs, the genes *RPH3AL*, *TPO*, and *PIWIL3* had CpG sites with largest changes in AVDM ranging from 12–13%. For the male offspring analysis, the CpG sites with the largest *AVDM* were assigned to *ERICH1-AS1*, *GSTM5*, and *SCML4* with *AVDM* ranging from 23–30%. *GSTM1* only possessed one differentially methylated CpG, but was of interest given its shared classification and close genetic proximity with *GSTM5* within the glutathione S-transferase family. For the female offspring analysis, CpGs assigned to *SEC16A*, *DECR1*, and *KCNE1* had the largest changes in *AVDM* ranging from 20–30%. *KCNE1* and *NXN* had 6 and 5 differentially methylated CpG sites, respectively. Validation of changes in DNA methylation by the Infinium450K Methylation Bead Array was performed with Mass Array Epityper and pyrosequencing. DNA methylation changes were in the same direction and of comparable magnitude with changes identified in the methylation array. Please see S1 Text and S1 Table.

**Table 2:** CpG sites with greatest absolute change in DNA methylation status*.

Group	Infinium Probe ID	Gene	Location	dm	p
All Pairs	cg06948435	RPH3AL	Chr17:123,982	0.1315	9.27E-04
	cg04392293	N/S	Chr11:28,399,978	0.1312	5.11E-04
	cg02197192	N/S	Chr 8:64,968,884	0.1303	6.95E-04
	cg07713008	TPO	Chr 2:1,516,247	0.1257	5.47E-04
	cg03438754	PIWIL3	Chr 22:25,171,162	0.1206	3.73E-04
	cg23248887	SSTR1	Chr 14:38,679,643	-0.1169	2.31E-04
	cg04670922	PTPRN2	Chr 7:157,527,872	0.1117	4.66E-04
	cg14743683	PTPRN2	Chr 7:157,872,788	-0.1109	6.95E-04
	cg24436207	PIWIL3	Chr 22;25,170,859	0.1108	9.80E-04
	cg20160351	DAB1	Chr 1: 58,555,364	0.1063	3.91E-04
Male Pairs	cg08270148	ERICH1 –AS1	Chr8: 914,819	0.3057	2.46E-04
	cg25593510	GSTM5	Chr1: 110,254,663	-0.2849	9.09E-04
	cg12858902	GSTM5	Chr1: 110,254,855	-0.2652	2.65E-04
	cg16656875	SCML4	Chr6:108,145,931	-0.2328	6.33E-04
	cg10950028	GSTM1	Chr1: 110,230,634	-0.2288	3.54E-04
	cg01078903	MIR487B	Chr14: 101,512,375	-0.2065	8.51E-04
	cg08919443	COL6A1	Chr21: 47,423,639	0.2037	7.87E-04
	cg22282405	TRAP2B	Chr6: 50,810,682	0.2024	5.24E-04
	cg04952609	N/S	Chr2: 139,664,238	-0.1944	3.20E-04
	cg02118671	N/S	Chr8: 143,660,413	0.1770	2.22E-04
Female Pairs	cg21243064	SEC16A	Chr9: 139,371,234	0.3067	7.15E-04
	cg06902669	DECR1	Chr8: 91,014,265	-0.2621	3.06E-04
	cg11872321	DECR1	Chr8: 91,014,327	-0.2049	2.73E-04
	cg23480619	KCNE1	Chr21: 35,831,872	0.2028	1.17E-04
	cg14535332	KCNE1	Chr21: 35,831,955	0.1972	2.83E-05
	cg13661012	TPO	Chr2: 1,516,352	0.1952	6.91E-04
	cg09187549	N/S	Chr1: 170,456,528	0.1821	4.42E-05
	cg15640734	SLC9A3	Chr5: 494,837	0.1608	5.28E-04
	cg18447419	SLC9A3	Chr5: 496,300	0.1594	4.68E-05
	cg17224775	N/S	Chr8: 2,483,326	0.1549	9.30E-04

* (p<0.001)

### RNA-Seq

**Fig 4:**
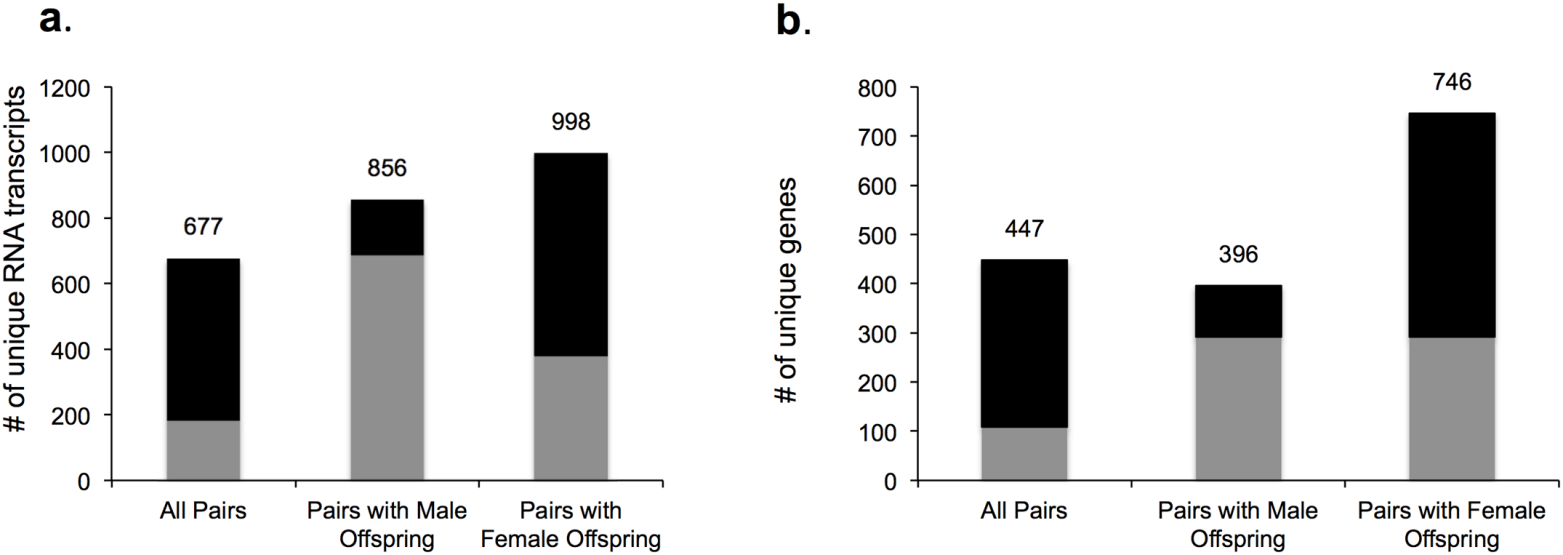
Differentially expressed genes in placenta exposed to DDP identified via RNA-Seq (n = 3 pairs per offspring sex). Black represents differentially expressed transcripts with control > DDP; Gray represents DDP> control. a. Differentially expressed unique transcripts. b. Differentially expressed unique genes. RNA transcripts with expression sum >5, fold change>1.5, and *p*-value < 0.05.

We found a limited number of genes identified with differential expression when using the sole criteria of q<0.05 using the Benjamini-Hochberg method to correct for multiple hypotheses testing (none in *all* pairs analysis, 2 in the female pair analysis and 10 in male pair analysis (Table 3). Therefore, RNA transcripts with expression sum >5, fold change>1.5, and *p*-value < 0.05 were considered to be differentially expressed between DDP and control for subsequent studies. In the analyses of *all* pairs, pairs with male offspring, and pairs with female offspring, a total of 677, 856, and 998 mRNA transcripts, respectively, showed significant changes in expression (Fig 4). These RNA transcripts corresponded to 447, 396 and 746 unique genes in the analysis of *all* pairs, pairs with male offspring, and pairs with female offspring respectively (Fig 4). S2 Table lists the 50 most differentially expressed mRNA transcripts ranked by *p*-value.

**Table 3:** Placental RNA transcripts identified by RNA-Seq with differential expression*.

Gene	q value	Fold change	p value	Function
**Female pair analysis**				
WIPF2	0.03	-1.3	0.0001	Encodes for Wiskott Aldrich Syndrome Protein (WASP) interacting protein (WIP)-related protein 2 which has a role in the WASP-mediated organization of the actin cytoskeleton.
PDCD6	0.05	-1.3	<0.0001	Encodes for a calcium-binding protein belonging to the penta-EF-hand protein family. This gene product participates in T cell receptor-, Fas-, and glucocorticoid-induced programmed cell death.
**Male pair analysis**				
ABCA5	0.003	-333.3	<0.0001	Encodes for ABC protein that transports molecules across extra- and intracellular membrane
TBL1XR1	0.01	-149.3	<0.0001	Encodes for protein that is a component of both nuclear receptor co-repressor (N-CoR) and histone deacetylase 3 (HDAC 3) complexes
CCNT1	0.01	-94.3	<0.0001	Encodes for a protein tightly associates with cyclin-dependent kinase 9, and is a major subunit of positive transcription elongation factor b (p-TEFb)
ITPR3	0.01	3.4	<0.0001	Encodes a receptor for inositol 1,4,5-trisphosphate, a second messenger that mediates the release of intracellular calcium
WNK1	0.03	1.4	<0.0001	Encodes for a member of the WNK subfamily of serine/threonine protein kinases and is a key regulator of blood pressure.
TOX4	0.03	-37.6	<0.0001	Encodes for a component of the PTW/PP1 phosphatase complex, which plays a role in the control of chromatin structure and cell cycle progression during the transition from mitosis into interphase
STAT6	0.03	1.7	<0.0001	Encodes for a protein with a central role in exerting IL4 mediated biological responses. It is found to induce the expression of BCL2L1/BCL-X(L), which is responsible for the anti-apoptotic activity of IL4.
ASB3	0.03	-30.7	<0.0001	Encodes for a protein that couples with suppressor of cytokine signaling (SOCS) proteins and their binding partners with the elongin B and C complex, possibly targeting them for degradation.
SRPK2	0.05	1.4	<0.0001	Encodes for a serine/arginine-rich protein-specific kinase which specifically phosphorylates its substrates at serine residues located in regions rich in arginine/serine dipeptides. Upregulates Cyclin D1.
FUS	0.05	-21.0	<0.0001	Encodes for a multifunctional protein component of the heterogeneous nuclear ribonucleoprotein (hnRNP) complex involved in pre-mRNA splicing and the export of fully processed mRNA to the cytoplasm

* q <0.05

### Protein abundance

**Fig 5:**
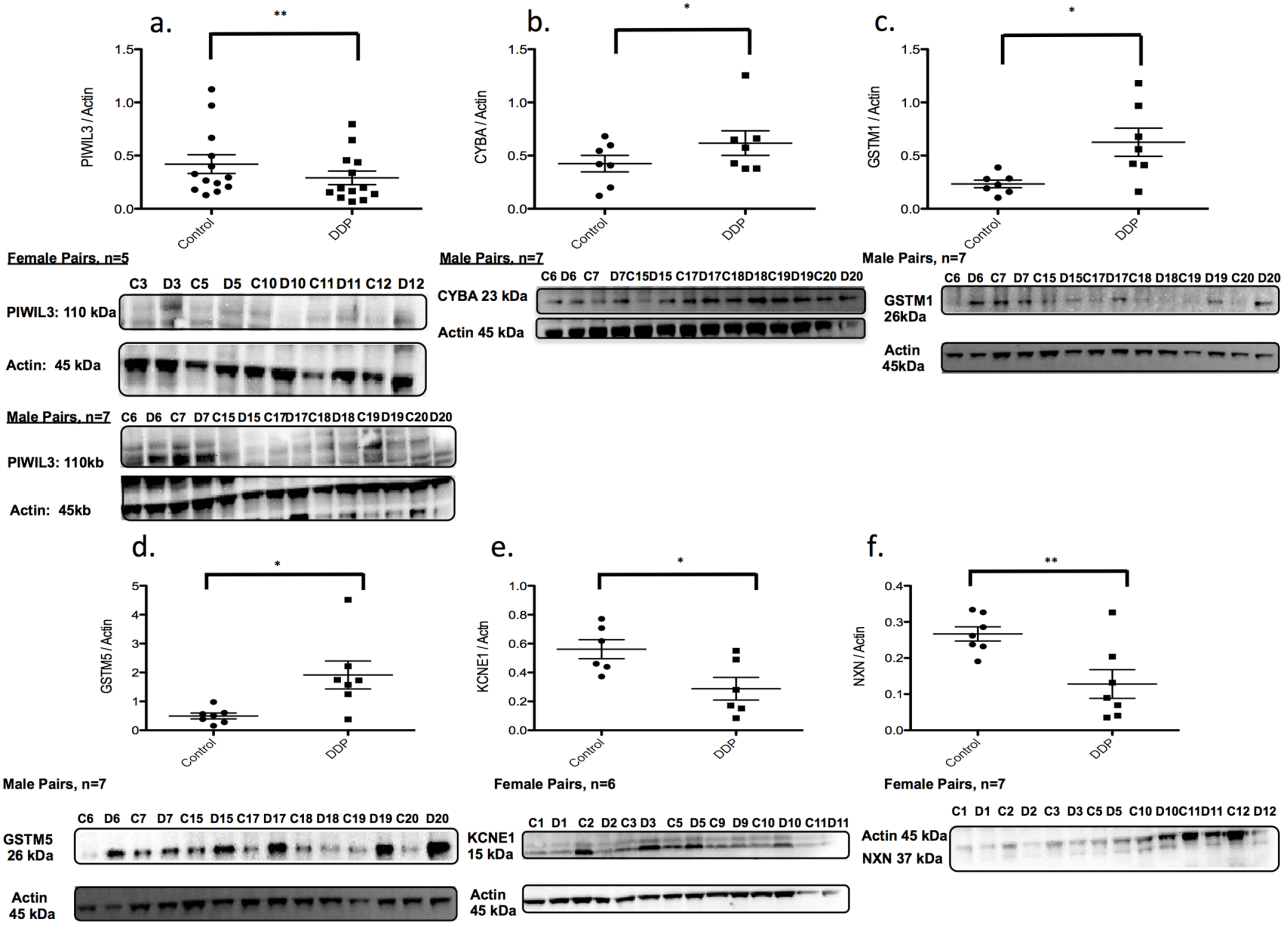
Protein abundance for genes with changes in DNA methylation and mRNA expression. a. PIWIL3 b. CYBA c. GSTM1 d. GSTM5 e. KCNE1 f. NXN; a–f: Protein abundance normalized to Actin or cofillin. a: *All* pairs (N = 14; 7 male pairs and 7 female pairs). b-d: Male offspring pairs (n = 7). e-f: Female offspring pairs (n = 7). * p<0.05; ** p<0.05.

We aimed to investigate if changes in DNA methylation were associated with downstream changes in mRNA expression and protein abundance. Therefore, we used data from the DNA methylation and RNA-Seq experiments with information about placental protein abundance and antibody performance to choose candidate genes for subsequent studies (Table 4, Fig 5, S3–S5 Tables). Protein isolated from placentae was assayed via Western Blot. In the analysis of *all* pairs, we found a 30% decrease in protein levels of PIWIL3 in DDP samples, which corresponded to a 30% decrease in mRNA levels and an 11–12% increase in DNA methylation at 2 CpG sites. PIWIL3 is an Argonaute protein involved in RNA silencing [18]. In placenta samples from male offspring, protein levels for GSTM1 and GSTM5 were increased 200–400%, corresponding to a 50% increase in mRNA expression and a decrease in DNA methylation of 9–28% at 5 CpG sites. GSTM1 and GSTM5 are glutathione-S-transferases involved in neutralizing and removing reactive oxygen species (ROS) [19]. CYBA protein levels were significantly increased 45% in DDP samples from male offspring pairs, which corresponded to an almost 200% increase in mRNA levels, and a 13–15% decrease in DNA methylation at 2 CpG sites. CYBA encodes for a protein that is part of the NADPH oxidase complex that generates ROS [20]. In protein isolated from placentae from female offspring pairs, KCNE1 and NXN were decreased in DDP samples 50%, again corresponding to a 50% reduction in mRNA levels and 9–20% increase in DNA methylation at 6 CpG sites for KCNE1 and 5–10% increase in DNA methylation at 5 CpG sites for NXN. KCNE1 is a delayed rectifier potassium channel and NXN is a member of the thioredoxin super family of proteins [21–23].

**Table 4:** Correlation between DNA methylation, RNA-Seq, and protein levels.

		DNA Methylation				RNA-Seq		Protein Levels		
	Gene	CpG	dm	CpG location	p	Fold Change	p	Fold Change	p	Gene Function
**All Pairs**	**PIWIL3**	cg24436207	0.11	promoter, weak enhancer	9.8E-04	-1.43	0.403	-1.45	0.013	PIWI Argonaute protein; RNA silencing
		cg03438754	0.12		3.7E-04					
**Male Pairs**	**CYBA**	cg26537639	-0.13	active promoter & CpG island	2.4E-04	1.96	0.253	1.45	0.043	Component of NADPH oxidase complex
		cg04879832	-0.15		2.1E-04					
	**GSTM1**	cg10950028	-0.23	promoter w/ CpG island	3.5E-04	6.49	0.389	2.68	0.027	Members of GST protein family that neutralize ROS and toxins to prevent DNA damage
	**GSTM5**	cg12858902	-0.27	promoter	2.7E-04	1.53	0.421	3.86	0.042	
		cg05376982	-0.15		6.3E-04					
		cg25593510	-0.28		9.1E-04					
		cg23719124	-0.09		2.0E-04					
**Female Pairs**	**KCNE1**	cg23480619	0.20	CpG island and poised promoter	1.2E-04	-2.15	0.528	-1.96	0.051	Potassium channel whose currents are suppressed by insulin
		cg08823027	0.09		8.4E-04					
		cg23908228	0.11		2.3E-04					
		cg07321776	0.13		1.3E-05					
		cg19521832	0.15		7.5E-04					
		cg14535332	0.20		2.8E-05					
	**NXN**	cg08192143	0.05	weak enhancer	3.0E-04	-1.86	0.060	-2.08	0.019	Redox-dependent regulator of Wnt signaling pathway; involved in cell growth and differentiation
		cg19431200	0.05		6.4E-04					
		cg20403644	0.06		5.7E-04					
		cg15019011	0.08		4.9E-04					
		cg24015037	0.10		4.3E-04					

We also aimed to measure how exposure to DDP affected mRNA and protein levels in placental genes without changes in DNA methylation. Candidate genes were selected based on the RNA-Seq data and information about placental protein abundance and antibody quality (Table 5, S1 Fig). Protein levels of ALG1 were decreased 36% in DDP samples (p = 0.0145) corresponding to a 50% decrease in mRNA expression in DDP samples from both male offspring. ALG1 encodes for beta 1,4 mannosyltransferase, an enzyme disrupted in a congenital disorder of glycosylation [24–26]. Protein levels of BCL2 and ARNT in DDP were decreased 28–29% in samples from male offspring only corresponding to a 20–35% decrease in mRNA (p = 0.0196 and = 0.056) while for SPRY1, protein levels were increased 58% in DDP and mRNA levels increased 52% (p = 0.009). BCL2 is an anti-apoptotic protein, SPRY1 prohibits cell proliferation and ARNT regulates placental angiogenesis [27–29]. Finally, protein levels of MTHFD1L were increased almost 70% in DDP samples from female offspring corresponding to an 84% increase in mRNA levels (p = 0.0104). MTHFD1L is an enzyme involved in synthesizing tetrahydrofolate, an critical factor in embryonic development [30].

**Table 5:** Genes with correlating RNA-Seq and protein levels.

		mRNA-seq			Protein Levels	
	gene	fold change	*p*	*q*	fold change	*p*
**Male**	ALG1	-2.00	1.88E-04	0.8173	-1.56	0.0145
	BCL2	-1.60	8.59E-04	0.0646	-1.64	0.0196
	SPRY1	1.52	1.89E-04	0.0957	1.58	0.009
	ARNT	-1.24	1.00E-04	0.1874	-1.61	0.056
**Female**	MTHFD1L	1.84	1.00E-04	0.2953	1.69	0.0104

### Ingenuity pathway analysis

IPA identified significantly enriched canonical pathways using lists of genes identified with significant differences in DNA methylation and significant changes in RNA expression between DDP and control samples. Separate analyses were performed comparing *all* samples, samples from male offspring and samples from female offspring. Genes identified with significant changes in DNA methylation had statistical enrichment in pathways related to mitochondrial function, DNA repair, inflammation, oxidative stress (S6 Table). In RNA-Seq analyses, IPA identified enriched pathways related to cell signaling, inflammation, hematopoiesis and fatty acid metabolism (S7 Table). In both the DNA methylation and the RNA-Seq IPA analyses, unique pathways were identified as enriched in the *all*, male and female offspring analyses providing further evidence that DDP exposure has sex specific effects on the offspring.

## Discussion

Although the negative effects of the aberrant intrauterine milieu from DDP exposure have been well established the molecular mechanisms are poorly understood. In our study, we utilized a unique nested case-control design to strengthen our ability to draw conclusions regarding biologically significant changes in DNA methylation, mRNA expression, and protein levels in placentae from diabetic pregnancies. Our results show significant changes in DNA methylation of genes related to metabolism, inflammation, autoimmunity, cell cycle, and cell death. These results were corroborated by corresponding patterns in RNA and protein levels for a subset of genes.

A major finding in our study was that sex of the offspring was associated with significant changes in DNA methylation of placentae exposed to DDP. Placentae exposed to DDP from male infants had a greater number of CpG sites with significantly altered DNA methylation. In addition, DDP exposure was associated with an increase in the proportion of CpG sites with a significant gain in DNA methylation in placentae from female offspring. Finally, separate analyses for placentae from male and female offspring found that DDP is more likely to alter DNA methylation at CpG sites within the first exon, but the specific CpG sites affected are heavily influenced by offspring sex since the effect is lost with the *all* pairs analysis. Previous studies have described sex of the offspring affecting placental gene expression and function, but the mechanisms responsible have yet to be elucidated [31]. The findings regarding sex specific DNA methylation patterns in the first exon are particularly interesting as the DNA methylation of this region has been tightly linked to transcriptional silencing [32]. The sex specific programming effects on the placenta are consistent with epidemiological studies that report sex specific changes in glucose homeostasis and insulin sensitivity in studies of pre-pubertal offspring exposed to DDP [33, 34].

In our analysis of *all* pairs, our most significant differentially methylated gene was *PIWIL3*, with two unique CpG sites affected (*AVDM* = 0.11 and *0*.12). *PIWIL3* is a member of the PIWI subfamily of Argonaute proteins and is involved in RNA-mediated gene silencing [18] which has effects on embryogenesis [35, 36]. Since *PIWIL3* is unique to mammals, this gene is particularly interesting in the context of fetal programing [36].

Additional analyses of male offspring placenta pairs identified three genes with decreased DNA methylation and corresponding increases in RNA and protein related to ROS processing. *CYBA*, which encodes for p22(phox), a component of the NADPH oxidase complex [20] that increases ROS contributing to placental dysfunction in DDP pregnancies [37–39]. GSTM1 and GSTM5 are members of the glutathione-S-transferases (GSTs) family of enzymes that neutralize and remove ROS to prevent DNA damage [19]. The reduction in DNA methylation and increased expression of GSTM1 and GSTM5 may be a compensatory mechanism to defend against increased ROS in DDP.

Two genes, *KCNE1* and *NXN*, were found to have increased DNA methylation and decreased mRNA expression and protein levels in DDP placentae from female offspring. KCNE1 is a potassium channel subunit whose currents are suppressed by insulin [23]. *NXN*, encodes nucleoredoxin, a member of the thioredoxin super family involved in cell growth and differentiation [22, 40]. NXN deficiency augments NADPH and reduces levels of glutathione [21] suggesting a compensatory mechanism in DDP to reduce ROS.

In the focused RNA-Seq analysis of *all* pairs, the two of the most differentially expressed transcripts were *ALG1*, which encodes beta 1,4 mannosyltransferase and *ALG9*, which encodes alpha 1,2 mannosyltransferase, both enzymes which have been implicated in congenital disorders of glycosylation [24–26]. A corresponding directional change in protein levels for Alg1 was confirmed via Western Blot. Additional studies have shown that lipid glycosylation and mannosyltransferase activity are upregulated in diabetic patients [41].

In RNA-Seq data from placentae with male offspring, *BCL2*, which encodes B-cell lymphoma 2, and *ARNT*, (also known as *HIF1-β*), which encodes the aryl hydrocarbon receptor nuclear translocator, were decreased in DDP placentae. Conversely, *SPRY1*, which encodes sprout RTK signaling antagonist 1, was increased in DDP placentae. BCL-2 is an anti-apoptotic protein, and there are established links between gestational diabetes, decreased placental BCL-2 expression and increased apoptosis in trophoblasts [27]. Previous studies have shown ARNT is critical to placental angiogenesis and decreased ARNT results in structurally abnormal placental vasculature in growth-restricted fetuses [28]. Our data indicate that decreased expression of ARNT may affect placental angiogenesis in DDP. In the placenta, SPRY proteins are important regulators of branching morphogenesis and growth factors signaling [29] and increased SPRY1 expression could contribute to unregulated growth in DDP placentae. These data support the findings of other studies showing that DDP impairs placental vascularization [42, 43]

In placentae from female offspring from women with DDP, we found increased mRNA and protein levels of *MTHFD1L*, which encodes formyltetrahydrofolate synthetase and is involved in the synthesis of tetrahydrofolate (THF) in the mitochondria [30]. Recently MTHFDL1 was reported to control DNA methylation in *Arabidopsis* [44], and if this finding holds true in mammalian studies, DDP induced changes in MTHFD1 expression could link DDP exposure to altered DNA methylation in placenta.

Our study was limited by the small samples size, which was likely a major factor as to why we were unable to detect significant changes after correction for multiple hypothesis testing. However, we were able to follow up the DNA methylation findings with both RNA-Seq and protein data for a number of metabolically relevant genes. We have the most confidence in the findings of genetic loci with changes in DNA methylation that correlate with changes in RNA-Seq and protein levels. Other limitations to our study include heterogeneity in the type of maternal diabetes (gestational vs. Type 2) and treatment modalities in the DDP group. In addition, the mothers in the control group had increased parity compared to DDP mothers and it is possible that the difference in parity may be responsible for the lack of difference in birth weight, which ultimately may have affected our results.

Several other studies have reported effects of maternal diabetes on genome wide DNA methylation of placentae but these studies focused on patients with gestational diabetes (not preexisting diabetes), and did not focus on Native American and Hispanic populations nor did they employ a nested case control design [45–49]. It is plausible that abnormalities in glucose control and insulin resistance in women during the first and early second trimester of pregnancy may have significant effects on placental and fetal development and differences in early gestation glucose and insulin homeostasis could account for the differences in affected gene targets in diabetes exposed placentas identified amongst similar studies. However in the present study, only 3 of 17 (18%) mothers with DDP had pre-existing diabetes prior to pregnancy. An alternative explanation for the differences in affected genes described in diabetes-exposed placentae from other cohorts could be due to gestational glycemic control. Given that the patients with DDP in the this study had well-controlled diabetes characterized by hgbA1C of 5.8%, which may account for the differences in genes affected by DNA methylation and expression that are described by other cohorts.

In our study, we did not detect significant changes in DNA methylation for LEP, ADIPOQ, VIPR1, PPARA, MEST, NRC31, IGF2 and H19 that were reported previously [45–49]. However, we were able to corroborate findings from several other studies. Rong et al report hypermethylation in the TPO gene included in Hematopoietic Cell Lineage, Jak-Stat signaling and Cytokine-Cytokine Receptor interaction pathways and this gene had a significant increase in DNA methylation in our *all* pairs analysis [50]. In addition, Rong et al report hypomethylation in the glutathione metabolism pathway, a pathway highlighted in our study with decreased DNA methylation measured at several loci associated with GSTM5 and GSTM1 [50]. Other studies report changes in DNA methylation in genes related to autoimmunity and the human leukocyte antigen (HLA) complex, which were also seen in our analyses [50, 51]. Finally, similar to our findings, Finer et al report that changes in DNA methylation are enriched in the first exon region and tied to transcriptional silencing [52]. Our results, as well as those described in other cohorts suggest that additional mechanistic studies are needed in animal models and human samples to elucidate the specific mechanisms responsible in the placenta for fetal programming effects after maternal diabetes exposure with the ultimate goal to develop strategies for prevention. In addition, ongoing studies are characterizing the metabolic profiles of the diabetes exposed whose placentas were described in this study.

In conclusion, we found that DDP alters placental DNA methylation at metabolically relevant loci in a sex specific manner, with more CpG sites affected in placentae from male offspring. Many of the differentially methylated genes had corresponding changes in both RNA expression and protein levels, while other genes were found to have differential RNA expression and protein levels despite no measurable change in DNA methylation. Furthermore, these changes were observed in the face of near optimal management of dysglycemia. These findings may begin to explain the long-term metabolic effects of diabetes during pregnancy on offspring, as the placenta is an essential regulator of fetal growth and development.

## Supporting information

S1 Text (DOCX)

S1 Table Validation studies for genome wide DNA methylation data. (DOCX)

S2 Table 50 top differentially expressed mRNA transcripts. (XLSX)

S3 Table Correlation between DNA methylation and RNA- Seq—all pairs analysis. (XLSX)

S4 Table Correlation between DNA methylation and RNA- Seq—male pairs analysis. (XLSX)

S5 Table Correlation between DNA methylation and RNA- Seq—female pairs analysis. (XLSX)

S6 Table Enriched canonical pathways—DNA methylation. (DOCX)

S7 Table Enriched canonical pathways—RNA-Seq. (DOCX)

S1 Fig Protein abundance for genes with differentially expressed mRNA transcripts. Protein abundance for genes with corresponding changes in mRNA expression but no change in DNA methylation. A. ALG1 B. BCL2 C. SPRY1 D. ARNT E. MTHFDL1 A—E: Protein abundance measured via densitometry and normalized to Actin or cofillin. A—D: Male offspring pairs (n = 7). E: Female offspring pairs (n = 7).
(TIFF)

## Abbreviations

AVDM: absolute value of the difference in methylation between DDP and control
BMI: body mass index
DDP: diabetes during pregnancy
GST: glutathione transferase
HLA: human leukocyte antigen
IPA: Ingenuity Pathway Analysis
ROS: reactive oxygen species
THF: tetrahydrofolate
