# Supplementary material for: Offspring sex impacts DNA methylation and gene expression in placentae from women with diabetes during pregnancy
Source: PLoS One. 2018 Feb 22;13(2):e0190698. doi: 10.1371/journal.pone.0190698 (PMC5823368; doi:10.1371/journal.pone.0190698)
Supplement: S1 Text — (DOCX) [file pone.0190698.s001.docx]

**S1 Text**

**Placental Dissection:** Placentae were dissected as soon as possible after delivery, generally within one hour. An approximately three cm diameter core was taken by cutting from the fetal surface down through the maternal surface in a location roughly halfway between the umbilical cord attachment site and the placental margin in an area free from any obvious abnormalities. The core was cut into thirds such that one-third was fetal-side tissue, one-third was maternal-side tissue and the middle third, containing the maternal-fetal interface, was discarded. The fetal membrane was removed and the remaining tissue washed with ice-cold saline, blotted dry and stored at -80°C. Only the fetal-side placenta samples were used in this analysis.

**RNA-Sequencing:** A) Sample QC, Library Preparation and Sequencing: Total RNA was isolated using RNeasy Mini Kit (Qiagen). RNA was quantified on the High Sensitivity RNA Qubit assay, and quality was assessed using the Agilent Bioanalyzer. Library preparation and high-throughput sequencing were performed by Cofactor Genomics (http://cofactorgenomics.com, Saint Louis, Missouri, USA). Total RNA was quantified using a Qubit RNA High Sensitivity Assay (Life Technologies). 200 ng of total RNA used for cDNA synthesis with random priming using the Nugen Ovation Human FFPE-RNA-Seq System (Cat #**0340-32)** and fragmented to an average size of 200 bp using the Covaris S2. Fragmented cDNA was end repaired, A-tailed and ligated with barcoded Illumina adapters. Final library yield was measured using the Qubit High Sensitivity DNA Qubit assay and library size assessed using the Agilent 2100 Bioanalyzer. The libraries were sequenced on an Illumina NextSeq 500 using single-end 75 bp reads. B) RNA-Seq Analysis including quality control, alignment, clustering, normalization, and expression comparison: Analysis and visualization were performed by Cofactor Genomics (http://cofactorgenomics.com, Saint Louis, Missouri, USA). Raw sequence data in FASTQ format were assessed for quality (FastQC, http://www.bioinformatics.babraham.ac.uk/projects/fastqc/) and ribosomal RNA content. NovoAlign (Novocraft, http://novocraft.com) was used to align reads to a set of transcript sequences and also to the reference genome. NovoAlign parameters were set to allow multiple alignments to the transcript set, but only unique alignments to the genome. The genome alignment loci from all samples were combined and clustered to generate genomic loci (“patches”) with contiguous read coverage. Patches overlapping reference genome annotation loci were annotated as such. For each transcript or patch, the mean coverage (number of bases of read sequence aligned to the transcript or patch divided by the length of the transcript or patch) was calculated for each sample and was then further normalized by multiplying each mean coverage by the mean number of aligned reads per sample divided by the number of aligned reads for that sample. This was the final per-sample expression value. For each replicate group, the mean and coefficient of variation for each transcript or patch were calculated across the expression values for the samples in that group. These means were considered to be the expression values for the replicate group. P-values were calculated between the means of each pair of replicate groups using a Welch’s t-test corrected for FDR by the method of Benjamini-Hochberg. The resulting comparative expression data were visualized in ActiveSite (Cofactor Genomics), and loci of interest were chosen.

**Bisulfite Pyrosequencing:**

One ug of genomic DNA was denatured in 0.3M NaOH for 10 min at 37°C before the addition of freshly prepared 3.5M sodium metabisulfite (Sigma USA) and 1 mM of hydroquinone solution (Sigma, USA) and incubated for 20 min at 50°C, 15s at 85°C for 48 cycles. Samples were desulfonated in 0.3M NaOH for 15 minutes at 37°C and neutralized DNA was precipitated overnight at -20°C in 1uL of glycogen (10mg/mL), 17 uL of ammonium acetate and 50uL of cold absolute ethanol. Samples were resuspended in 20uL of DEPC H20 after precipitation. Sodium bisulfite modified genomic DNA was amplified using Hot-Start Taq master mix (Qiagen) as previously described. Regions of interest were amplified by PCR and pyrosequencing was carried out using the PyroMark Q24 pyrosequencer (Qiagen) according to the manufacturer's protocol (PyroGold reagents). Output data were analyzed using PyroMark Q24 1.0.10 Software (Qiagen), which calculates the methylation percentage (mC/(mC+C)) for each CpG site, allowing quantitative comparisons. Percent methylation was measured at each CpG site within the assayed region and averaged across the entire region for each sample and dm was calculated for each paired sample. A paired two-sample t-test was performed to estimate the methylation change and statistical significance for each assayed region.

**GSTM5 primers:**

**Probe 1:**

F1: AGGGTTGGATGGATTTATGG

R1: AACTAAAATCAATAAACCCCCTCC

PCR: TGGATGGATTTATGGAG

**Probe 2:**

F1: GGAGGGGGTTTATTGATTTTAG

F2: CCCAATACCCCAAAATCATAAACATAATA

PCR:GGGAGGGGTAGTAGTAAGGTT

**Mass Array Epityper:** MassArray EpiTyper analysis was performed on selected regions of genes identified with more than one Illumina Probe site with significant change in methylation between DDP and control placentae and within close proximity including RASSF2, CYBA, DECR1, amd KCNE1. Genomic DNA was bisulfite converted as described above. Primers were designed with the Epidesigner tool and are listed below. EpiTYPER assays were done by the Weill Cornell Medical College Epigenomics Core (New York, NY). Percent methylation was measured at each CpG site within the assayed region and averaged across the entire region for each sample and dm was calculated for each paired sample. A paired two-sample t-test was performed to estimate the methylation change and statistical significance for each assayed region.

**Primers:**

**CYBA:**

**F:** aggaagagagGTAAGGGAATTATTGGGGGTTTTA

R: cagtaatacgactcactatagggagaaggctAAACAACCCTACACCCTACAAATAC

**RASSF2**

**F:** aggaagagagTTGTTTGTTTTGGGTATAGTTGATT

R: cagtaatacgactcactatagggagaaggctACAACTCATCACACAAACATTTAAAAC

**KCNE1**

F: aggaagagagGGAGGTGGGAGATTTTAATTTAT

R: cagtaatacgactcactatagggagaaggctCCTTTCTCCTAACTACAATAACTTCCC

**DECR1**

**F:** ggaagagagGGTAGGAAGTTTTGGGGTATAAAGA

R: cagtaatacgactcactatagggagaaggctAAATAAACTTTCCTCACAACAAAAA
