# Supplementary material for: Offspring sex impacts DNA methylation and gene expression in placentae from women with diabetes during pregnancy
Source: PLoS One. 2018 Feb 22;13(2):e0190698. doi: 10.1371/journal.pone.0190698 (PMC5823368; doi:10.1371/journal.pone.0190698)
Supplement: S1 Table — (DOCX) [file pone.0190698.s002.docx]

**S1 Table: Validation studies for genome wide DNA methylation data**

| **Gene** | **Infinium 450 K Methylation Array** | | | **Confirmation Studies** | | | | |
| --- | --- | --- | --- | --- | --- | --- | --- | --- |
|  | **Probe(s)** | **dm** | ***p value*** | **Assay** | **dm** | ***p value*** | **#CpGs** | **Amplicon**  **Length** |
| GSTM5 | cg25593510  cg12858902 | -0.29  -0.27 | 9.09e-04  2.65e-04 | Bisulfite Pyrosequencing | -0.09 | 1.015e-5 | 2 | 142  210 |
| RASSF2 | cg12389461  cg03605116  cg03519577 | -0.14  -0.12  -0.09 | 0.0001  0.0005  0.0009 | Mass Array Epityper | -0.17 | 0.003 | 16 | 482 |
| CYBA | cg26537639  cg04879832 | -0.13  -0.15 | 0.0002  0.0002 | Mass Array Epityper | -0.08 | 0.032 | 34 | 419 |
| DECR1 | cg06902669  cg11872321 | -0.26  -0.21 | 3.06e-04  2.73e-04 | Mass Array Epityper | -0.14 | 0.04 | 19 | 364 |
| KCNE1 | cg23480619  cg08823027  cg23908228  cg07321776  cg19521832  cg14535332 | 0.20  0.09  0.12  0.13  0.15  0.20 | 0.00012  0.00084  0.00023  1.27e-05  0.00075  2.83e-05 | Mass Array Epityper | 0.075 | 0.03 | 32 | 448 |
