# Supplementary material for: Offspring sex impacts DNA methylation and gene expression in placentae from women with diabetes during pregnancy
Source: PLoS One. 2018 Feb 22;13(2):e0190698. doi: 10.1371/journal.pone.0190698 (PMC5823368; doi:10.1371/journal.pone.0190698)
Supplement: S6 Table — (DOCX) [file pone.0190698.s007.docx]

**S6 Table: Enriched canonical pathways - DNA methylation**

| **All Pairs  *n=346** | | | **Male Offspring Pairs  n=465** | | | **Female Offspring Pairs  n=340** | | |
| --- | --- | --- | --- | --- | --- | --- | --- | --- |
| **Pathways** | **p** | **genes** | **Pathways** | **p** | **genes** | **Pathways** | **p** | **genes** |
| Estrogen Receptor Signaling | 1.95E-03 | 7 | dTMP De Novo Biosynthesis | 1.95E-03 | 2 | Tec Kinase Signaling | 1.20E-04 | 9 |
| Aryl Hydrocarbon Receptor Signaling | 1.29E-02 | 6 | Coagulation System | 1.32E-02 | 3 | Rac Signaling | 1.58E-03 | 6 |
| Lipid Biosynthesis | 1.32E-02 | 2 | RAN Signaling | 2.09E-02 | 2 | Signaling by Rho Family GTPases | 2.14E-03 | 9 |
| PTEN Signaling | 2.34E-02 | 5 | MSP-RON Signaling Pathway | 2.69E-02 | 3 | Thrombin Signaling | 2.14E-03 | 8 |
| Valine Degradation I | 2.51E-02 | 2 | L-DOPA Degradation | 2.82E-02 | 1 | Phospholipase C Signaling | 2.29E-03 | 9 |
| Autoimmune Thyroid Disease Signaling | 2.63E-02 | 3 | nNOS Signaling in Neurons | 2.88E-02 | 3 | Actin Nucleation by ARP-WASP Complex | 4.57E-03 | 4 |
| GDP-L-fucose Biosynthesis II (from L-fucose) | 2.75E-02 | 1 | Molecular Mechanisms of Cancer | 3.55E-02 | 10 | NFAT in Regulation of the Immune Response | 4.57E-03 | 7 |
| GADD45 Signaling | 2.75E-02 | 2 | Endothelin-1 Signaling | 3.55E-02 | 6 | RhoGDI Signaling | 4.90E-03 | 7 |
| DNA damage-induced 14-3-3σ Signaling | 2.75E-02 | 2 | G Beta Gamma Signaling | 3.63E-02 | 4 | Ephrin Receptor Signaling | 5.13E-03 | 7 |
| Thyroid Hormone Biosynthesis | 4.07E-02 | 1 | Production of NO and ROS in Macrophages | 4.27E-02 | 6 | Relaxin Signaling | 5.75E-03 | 6 |
