# Supplementary material for: Offspring sex impacts DNA methylation and gene expression in placentae from women with diabetes during pregnancy
Source: PLoS One. 2018 Feb 22;13(2):e0190698. doi: 10.1371/journal.pone.0190698 (PMC5823368; doi:10.1371/journal.pone.0190698)
Supplement: S7 Table — (DOCX) [file pone.0190698.s008.docx]

**S7 Table. Enriched canonical pathways - RNA-Seq**

| **All Pairs  *n=447** | | | **Male Offspring Pairs n=396** | | | **Female Offspring Pairs  n=746** | | |
| --- | --- | --- | --- | --- | --- | --- | --- | --- |
| **Pathways** | **p** | **genes** | **Pathways** | **p** | **genes** | **Pathways** | **p** | **genes** |
| Heme Biosynthesis | 8.91E-04 | 2 | IL-10 Signaling | 7.94E-05 | 7 | Anaphase Transition and Mitotic Exit | 1.20E-03 | 7 |
| Protein Kinase A Signaling | 1.07E-03 | 12 | Tec Kinase Signaling | 1.55E-04 | 10 | HIPPO signaling | 1.29E-03 | 8 |
| Thrombopoietin Signaling | 4.68E-03 | 4 | Epithelial Adherens Junction Signaling | 4.17E-04 | 9 | Protein Ubiquitination Pathway | 1.78E-03 | 15 |
| Caveolar-Mediated Endocytosis | 4.90E-03 | 5 | Interferon-α/β Signaling | 4.47E-04 | 4 | Superpathway of Inositol Phosphate Compounds | 3.16E-03 | 12 |
| Dendritic Cell Maturation | 6.46E-03 | 7 | Death Receptor Signaling | 5.25E-04 | 7 | Cholesterol Biosynthesis | 3.55E-03 | 3 |
| G Protein Signaling Mediated by Tubby | 7.08E-03 | 3 | IL-15 Production | 7.08E-04 | 4 | Tight Junction Signaling | 8.71E-03 | 10 |
| Erythropoietin Signaling | 9.33E-03 | 4 | PI3K Signaling in B Lymphocytes | 7.59E-04 | 8 | Synaptic Long Term Depression | 8.91E-03 | 9 |
| Macropinocytosis Signaling | 1.00E-02 | 4 | JAK/Stat Signaling | 8.13E-04 | 6 | Wnt/β-catenin Signaling | 9.55E-03 | 10 |
| CCR5 Signaling in Macrophages | 1.05E-02 | 4 | IL-9 Signaling | 1.74E-03 | 4 | Cell Cycle Regulation by BTG Family Proteins | 1.05E-02 | 4 |
| Lanosterol Biosynthesis | 1.23E-02 | 1 | Interferon-γ Signaling | 2.14E-03 | 4 | Role of CHK Proteins in Cell Cycle Checkpoint Control | 1.15E-02 | 5 |
