# Supplementary figures and images for: Offspring sex impacts DNA methylation and gene expression in placentae from women with diabetes during pregnancy
Source: PLoS One. 2018 Feb 22;13(2):e0190698. doi: 10.1371/journal.pone.0190698 (PMC5823368; doi:10.1371/journal.pone.0190698)

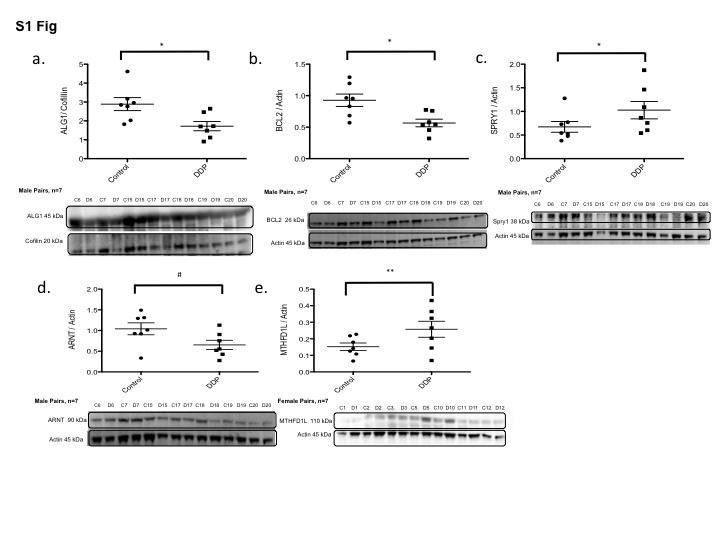

Supplement: S1 Fig — Protein abundance for genes with corresponding changes in mRNA expression but no change in DNA methylation. A. ALG1 B. BCL2 C. SPRY1 D. ARNT E. MTHFDL1 A—E: Protein abundance measured via densitometry and normalized to Actin or cofillin. A—D: Male offspring pairs (n = 7). E: Female offspring pairs (n = 7). (TIFF) [file pone.0190698.s009.tiff]
